# P-1604. Utilization of MRSA Nasal PCR to De-escalate Vancomycin Therapy in Inpatients with Suspected Pneumonia

**DOI:** 10.1093/ofid/ofae631.1771

**Published:** 2025-01-29

**Authors:** Jason Hedvat, Mudita Patel, Anjali Anne Ajit, Elisabeth L Rosen, David C Perlman, Sanjana Koshy

**Affiliations:** Mount Sinai Beth Israel, New York, New York; Icahn School of Medicine at Mount Sinai/Mount Sinai Beth Israel, New York, New York; Mount Sinai Morningside-West-Beth Israel, New York, New York; Mount Sinai Beth Israel, New York, New York; Icahn School of Medicine at Mount Sinai, New York, New York; Mount Sinai Beth Israel, New York, New York

## Abstract

**Background:**

MRSA nasal screening has been proposed as a tool to facilitate de-escalation of vancomycin in patients with pneumonia. The objective of this study was to evaluate the impact of MRSA nasal PCR testing in patients with pneumonia in terms of length of anti-MRSA therapy and clinical outcomes.

**Methods:**

This was a retrospective single center pilot study conducted at Mount Sinai Beth Israel during January 1, 2022 – March 31, 2022 (pre-availability of MRSA nasal PCR) and August 1, 2023 – October 31, 2023 (post-availability of MRSA nasal PCR). The primary outcome was the duration of vancomycin therapy in days in the pre-PCR compared to the post-PCR group. Secondary outcomes included the number of vancomycin levels drawn, 30- and 90- day mortality, hospital length of stay, and incidence of new acute kidney injury (AKI) during treatment. Comparisons were performed using Chi-squared or Fischer’s exact test for categorical variables and Student’s t test or the Mann-Whitney U test for continuous variables, as appropriate. P < .05 was considered statistically significant.

**Results:**

One-hundred and eighty-five patients were included, 91 in the pre-PCR group and 94 in the post-PCR group. The most common pneumonia indication was community acquired pneumonia (CAP) in 78% of patients. Among patients that received vancomycin therapy for CAP, only 30% (21/70) and 17.3% (13/75) had risk factors warranting anti-MRSA therapy in the pre-PCR and post-PCR groups respectively. The median duration of vancomycin therapy was significantly shorter in the post-PCR group than the pre-PCR group (0.9 days vs. 1.8 days; p < .05). Fewer patients required 2 or more vancomycin levels drawn in the post-PCR versus the pre-PCR group (20.2% vs 41.8%; p < .05). 30-day mortality was lower in the post-PCR group (12.8% vs 25.3%), but not significantly (p=0.08). No differences were found in other clinical outcomes including 90- day mortality, hospital length of stay, and incidence of new AKI during treatment.

**Conclusion:**

MRSA nasal PCR testing resulted in shorter duration of vancomycin therapy and reduced drug level monitoring without compromising clinical outcomes in patients with suspected pneumonia. Additional education is needed to ensure appropriate utilization of empiric anti-MRSA therapy in CAP patients with risk factors.

**Disclosures:**

**All Authors**: No reported disclosures

